# Role of Antiplatelets and Anticoagulation Therapies in Pregnancy

**DOI:** 10.3390/jcm13247757

**Published:** 2024-12-19

**Authors:** Krista A. Zachariah, Su Yuan, Maria T. DeSancho, Inna V. Landres, Harsimran S. Singh

**Affiliations:** 1 Division of Cardiology, Department of Medicine, NewYork-Presbyterian Hospital-Weill Cornell Medicine, New York, NY 10021, USA; kav9064@nyp.org (K.A.Z.); suy9028@med.cornell.edu (S.Y.); 2 Division of Hematology and Medical Oncology, Department of Medicine, NewYork-Presbyterian Hospital-Weill Cornell Medicine, New York, NY 10021, USA; mtd2002@med.cornell.edu; 3 Department of Obstetrics and Gynecology, NewYork-Presbyterian Hospital-Weill Cornell Medicine, New York, NY 10021, USA; ivl9003@med.cornell.edu

**Keywords:** antiplatelets, vitamin K antagonist, anticoagulation, hypercoagulable disorders, pregnancy, preeclampsia, thrombophilia, valvular heart disease, thrombosis, venous thromboembolism, mechanical valve, congenital heart disease

## Abstract

Thrombosis is an important cause of morbidity and mortality worldwide. Pregnancy is a hypercoagulable state, and thrombotic complications in pregnancy are a major cause of maternal and fetal morbidity and mortality. Current guidelines support the selective use of aspirin, heparin, and warfarin in pregnant women. The decision to treat with antiplatelets and anticoagulants during pregnancy can be challenging, as these agents have numerous indications and contraindications, potential teratogenicity, and dosing considerations. Their use requires thoughtful discussion between patient and provider that balances therapeutic benefit versus maternal and fetal risks, while accounting for a safe delivery plan. Our aim is to provide a narrative review of the established and emerging indications of antiplatelets and anticoagulants, discuss their appropriate timing of administration, and consider their associated adverse fetal and maternal risks.

## 1. Introduction

Thrombosis is an important cause of morbidity and mortality worldwide and has led to prioritization of the development and use of antiplatelet and anticoagulant agents. Antiplatelets inhibit clot formation by preventing platelet activation and aggregation, whereas anticoagulants inhibit the coagulation cascade and prevent thrombus formation by reducing fibrin formation. The most common indication for antithrombotic agents is for management of established cardiovascular, peripheral vascular, and cerebrovascular disease.

In the past several decades, there has been an increase in both thrombotic complications and overall maternal complications in women during pregnancy [1]. Some of the increase in disease incidence can be attributed to changes in population profile: increasing maternal age at first pregnancy; increasing prevalence of cardiovascular risk factors such as diabetes, obesity, and hypertension; and increasing numbers of women with moderate to complex congenital heart disease who are surviving to adulthood and proceeding with pregnancy [1]. Consequently, antiplatelet and anticoagulation therapies during pregnancy have become much more common place. It is crucial for providers caring for these patients to understand the benefits and risks specific to this clinical context.

Pregnancy is inherently a hypercoagulable state that results from increased levels of coagulation factors I (fibrinogen), VII, and VIII; von Willebrand factor; and factor X. Pregnant women also experience decreased free protein S (a natural anticoagulant), increased acquired resistance to activated protein C, and decreased fibrinolysis, owing to increased levels of plasminogen activator inhibitors 1 and 2, as well as increased D-dimer levels [2,3,4]. It is thought that hypercoagulability develops in pregnancy to protect women from major hemorrhage at the time of miscarriage or childbirth [4]. The risk of venous thromboembolism (VTE) in pregnancy is increased five-fold and continues into the postpartum period [2]. Therefore, thrombotic complications in pregnancy are a major cause of morbidity and mortality for both the mother and the child.

The risk of bleeding is greatest during the delivery period, and long-acting anticoagulant agents are problematic during delivery due to the potential need for neuraxial anesthesia or cesarean delivery [3]. This requires a delicate balance between minimizing bleeding risk and protecting against thrombosis. Recommendations for antithrombotics in pregnancy are derived largely from the nonpregnant population, and there are no large randomized controlled trials studying fetal safety in this setting. Current guidelines support the selective use of aspirin, heparin, and warfarin in pregnant women, with specific indications. The decision to treat with antiplatelets and anticoagulants during pregnancy can be challenging, as these agents have numerous indications and contraindications, potential teratogenicity, and dosing considerations. Their use requires thoughtful discussion between patient and provider that balances therapeutic benefit versus maternal and fetal risks, while accounting for a safe delivery plan. The aim of our study is to provide a narrative review of the established and emerging indications of antiplatelets and anticoagulants, discuss their appropriate timing of administration, and highlight their associated adverse fetal and maternal risks. 

## 2. Pathophysiology of Common Antiplatelets and Anticoagulants

Antiplatelets are often indicated during pregnancy, with the most clinical experience available for aspirin and clopidogrel. Aspirin is a cyclooxygenase inhibitor with both anti-inflammatory and antiplatelet properties. Aspirin modulates platelet function by the permanent acetylation of platelet cyclooxygenase [2]. It is a nonsteroidal anti-inflammatory drug that primarily inhibits COX-1 and COX-2, which are necessary for prostaglandin synthesis, in a dose-dependent fashion. At lower doses, aspirin irreversibly acetylates COX-1, resulting in decreased platelet synthesis of thromboxane A2 (TXA2), which promotes platelet aggregation and is a potent vasoconstrictor. At higher doses, aspirin inhibits both COX-1 and COX-2, which effectively inhibits all prostaglandin production [5]. Aspirin is the antiplatelet agent with the most safety data in pregnancy [2,5].

Clopidogrel is a P2Y_12_ inhibitor that irreversibly inhibits platelet aggregation and activation by preventing the binding of fibrinogen to its adenosine disphosphate receptor [2]. Other P2Y_12_ inhibitors include ticagrelor, prasugrel, and cangrelor (only available as an intravenous infusion). There are some safety data for clopidogrel use in pregnancy; there are none for the other agents. Ticlopidine is no longer available due to significant hematologic side effects, such as agranulocytosis, thrombotic thrombocytopenic purpura, and aplastic anemia.

The third class of antiplatelets are the glycoprotein IIb/IIIa (GP IIb/IIIa) inhibitors, which include tirofiban, eptifibatide, and abciximab. These agents inhibit the platelet surface GP IIb/IIIa receptor and fibrinogen binding. They are primarily used in acute coronary syndrome, around the time of percutaneous coronary intervention, and are given intravenously. There are no safety data for these agents in pregnancy.

Anticoagulants commonly used in pregnancy include vitamin K antagonists, heparin-based anticoagulants, and non-heparin anticoagulants. Warfarin is an oral vitamin K antagonist (VKA) that crosses the placenta. It produces an anticoagulant effect by interfering with the cyclic interconversion of vitamin K and its 2,3 epoxide (vitamin K epoxide). Vitamin K is required for γ-carboxylation of proteins, including coagulation factors II, VII, IX, and X. By inhibiting the vitamin K conversion cycle, warfarin induces hepatic production of partially decarboxylated proteins with reduced coagulant activity [6]. Warfarin is monitored with the international normalization ratio (INR), for which the general therapeutic goal range is between 2.0 and 3.5 [2].

Exposure to warfarin in the first trimester is associated with warfarin embryopathy, which is characterized by stippled epiphyses and nasal bone hypoplasia [2,7]. Exposure to warfarin in the third trimester is a risk factor for fetal intracranial hemorrhage during vaginal delivery. The risk of warfarin embryopathy appears to be dose-dependent: maternal daily doses of <=5 mg/day are associated with a <3% risk of warfarin embryopathy, compared to a >30% risk of fetal loss or embryopathy at maternal doses of over 5 mg/day [8]. To reduce the risk of warfarin embryopathy, guidelines recommend women on warfarin perform frequent pregnancy testing, with a full risk benefit discussion between patient and provider regarding when to switch between warfarin and other agents versus continuing warfarin through pregnancy [9]. This is discussed in detail in Section 4.3.

Heparin-based anticoagulants include unfractionated heparin (UFH) and low-molecular-weight heparin (LMWH). Both are used for the prevention or treatment of VTE in pregnancy. Neither UFH nor LMWH cross the placenta, and both are safe during lactation. LMWH consists of fragments of UFH produced by controlled enzymatic or chemical depolymerization processes. They differ in that UFH preferentially inhibits factor IIa (thrombin), potentiating the effect of antithrombin, while LMWH affects mainly factor Xa and, to a lesser extent, factor IIa [10]. The activated partial thromboplastin time (aPTT) is the main test used to monitor UFH. However, in the context of pregnancy, anti-Xa level is a better test, as the aPTT will be shortened due to increases in factor VIII in pregnancy. Pregnant patients using LMWH are recommended to have anti-Xa levels checked weekly to achieve a peak 0.7–1.2 U/mL and trough > 0.6 U/mL [7,11]. However, concerningly, thrombotic events have still occurred in patients who achieved therapeutic peak anti-Xa levels, possibly because peak anti-Xa levels may not be adequate to ascertain therapeutic anticoagulation. Discrepancies exist between different laboratory tests designed to measure levels of anticoagulation, as divergent values of anti-Xa and aPTT have been observed. The challenge lies in the fact that there is no functional test for monitoring the anticoagulant effect of LMWH [3]. LMWH has a longer half-life and dosing is weight-based. The two most common formulations available in the United States are enoxaparin and dalteparin [2]. UFH is preferred in cases of severe renal insufficiency.

Non-heparin anticoagulants include direct and indirect oral or parenteral Xa inhibitors, direct thrombin inhibitors, and heparinoids. These agents may be useful in patients with a history of heparin-induced thrombocytopenia (HIT) or heparin allergy. Fondaparinux is a synthetic pentasaccharide that indirectly inhibits factor Xa and is recommended by the American College of Obstetrics and Gynecology (ACOG) for anticoagulation in the setting of HIT or heparin allergy [2].

Direct oral anticoagulants (DOACs) are routinely used in clinical practice for anticoagulation in the non-pregnant population for prevention of thrombosis in several cardiovascular contexts, including lowering stroke risk and embolism in atrial fibrillation, as well as treatment of deep vein thrombosis and pulmonary embolism treatment. Commonly used DOACs include rivaroxaban, apixaban, dabigatran, and edoxaban [12]. The safety profile of DOACs in the non-pregnant population has been shown to be favorable, given generally lower bleeding risk when compared to VKAs [13,14]. However, DOACs are not approved for use in pregnancy [15]. Small retrospective cohort studies highlighting DOAC use in pregnancy are available, and pre-clinical evidence exists of placental transfer with the direct Xa inhibitors rivaroxaban and apixaban, and the oral thrombin inhibitor dabigatran, thus increasing the risk to the fetus [16,17].

## 3. Role of Antiplatelets in Pregnancy

### 3.1. Aspirin as Primary Prevention

Aspirin is generally indicated for women with bioprosthetic valves or a history of vascular event, such as myocardial infarction, coronary stent, or stroke. The most common indication for starting aspirin in pregnancy is to prevent or delay onset of preeclampsia [5], which is a major cause of maternal morbidity and mortality. Preeclampsia is a complex, multisystem inflammatory syndrome that is thought to result from early changes in placental development that lead to placental ischemia, inflammation, and oxidative stress. Preeclampsia may also develop due to overactive inflammatory responses to normal placentation. Initial studies to support aspirin use in preeclampsia were based on the hypothesis that preeclampsia is associated with vascular disturbances and coagulation defects that result from an imbalance between prostacyclin and TXA2 [5]. Subsequent randomized control trials confirmed this hypothesis and found that low-dose aspirin prophylaxis was associated with a 24% reduction in relative risk of preeclampsia, although the true risk reduction may be closer to 10% due to the “small study effect” in included trials [5].

Guidelines in the United States recommend starting low-dose aspirin prophylaxis (81 mg/day) between 12 and 28 weeks of gestation (optimally before 16 weeks) and continuing daily through delivery for women with an elevated risk of preeclampsia. The Combined Multimarker Screening and Randomized Patient Treatment with Aspirin for Evidence-Based Preeclampsia Prevention (ASPRE) trial demonstrated that aspirin, when initiated between 11 and 14 weeks, reduced the risk of preterm preeclampsia [18], where this specific timing is thought to align with early placental development stages before the uterine spiral arteries remodel, which can influence blood flow and endothelial function, which are both crucial to preventing preeclampsia [19]. The proposed mechanisms of aspirin’s effect include the direct effect on apoptosis proliferation of trophoblast cells, as well as antiplatelet prevention of placental infarctions [19].

Low-dose aspirin is recommended for women with one or more high-risk factors and should be considered for women with one or more moderate-risk factors (Table 1). High-risk factors for preeclampsia include history of preeclampsia, especially when accompanied by an adverse outcome; multifetal gestation; chronic hypertension; type 1 or 2 diabetes; renal disease; and autoimmune disease. Moderate-risk factors for preeclampsia include nulliparity, obesity, family history of preeclampsia, sociodemographic characteristics (African American race, low socioeconomic status), age 35 years or older, and personal history factors (e.g., low birthweight or small size for gestational age, previous adverse pregnancy outcome, interpregnancy interval of more than 10 years). Of note, European guidelines also suggest that women identified as high risk should receive aspirin prophylaxis starting at 11–14 weeks gestation but at a dose of 150 mg nightly until 36 weeks of gestation, when delivery occurs, or when preeclampsia is diagnosed [20]. Currently, there is no evidence for low-dose aspirin in pregnancy for women with a history of stillbirth, fetal growth restriction, preterm birth, or early pregnancy loss [5,21]. Aspirin is also indicated in certain cases of adult congenital heart disease, including those with unrepaired shunt lesions (for prevention of paradoxical embolism), aortic coarctation (due to increased risk of preeclampsia), and single-ventricle physiology (to prevent thromboembolism) [22]. Anecdotally, there is generally a lower threshold to offer aspirin to patients with moderate–complex adult congenital heart disease who are not already on anticoagulation.

Most recent trials show that the dose of aspirin used was 81–150 mg/day, but higher dosages of aspirin (100–150 mg/day) were associated with a greater reduction in risk of the onset of preeclampsia. Up to one-third of pregnant women proved to be aspirin-resistant at a dose of aspirin of 81 mg. Therefore, the recent consensus statement from the European Society of Hypertension now recommends 100–150 mg of aspirin once daily, preferentially at bedtime, as the preventive measure in women at high or moderate risk of preeclampsia, starting between 12 and 16 weeks of gestation [19].

### 3.2. Contraindications to Aspirin Use in Pregnancy

There are few contraindications to aspirin use in pregnancy. Patients with a history of aspirin allergy or hypersensitivity to other salicylates and/or nonsteroidal anti-inflammatory drugs (NSAIDs) are recommended to avoid aspirin. Patients with a history of nasal polyps should also avoid aspirin due to the risk of life-threatening bronchoconstriction. Finally, patients with asthma with a history of aspirin-induced acute bronchospasm should avoid aspirin for a similar reason. Relative contraindications to aspirin include history of gastrointestinal bleeding, active peptic ulcer disease, other sources of gastrointestinal or genitourinary bleeding, and severe hepatic dysfunction [5].

### 3.3. Maternal and Fetal Risks of Aspirin Use in Pregnancy

Risks of aspirin use in pregnancy are minimal. Numerous studies have demonstrated that low-dose aspirin is not associated with an increase in antepartum or postpartum hemorrhagic complications, including placental abruption, postpartum hemorrhage, or mean blood loss during delivery, or neonatal intracranial hemorrhage [5]. Aspirin has also not been associated with increased risk of congenital fetal anomalies [5]. There were a few studies that suggested a two-fold risk of gastroschisis in children of women using aspirin during pregnancy. However, these results were complicated by recall bias and several uncontrolled study variables, including the use of illicit and licit drugs. Furthermore, older studies suggested a relationship between in utero exposure to NSAIDs with premature closure of the ductus arteriosus and persistent pulmonary hypertension in the neonate but did not differentiate between the type and dose of NSAID exposure. More recent randomized controlled trials comparing aspirin use to placebo in over 30,000 women did not find an increase in perinatal deaths from persistent pulmonary hypertension in women who used aspirin [5].

### 3.4. Other Antiplatelets Used in Pregnancy

One particular indication for antiplatelet use in pregnancy is acute myocardial infarction. Pregnancy-associated myocardial infarction accounts for over 20% of maternal cardiac deaths and is defined as myocardial infarction during pregnancy or the postpartum period, typically from 6 to 12 weeks postpartum [23]. Acute myocardial infarction is reported in 1 per 10,000 pregnancies and occurs most commonly during delivery or the puerperium period in women over the age of 30. Aspirin has the most evidence for use compared to other antiplatelets. Clopidogrel is a reasonable substitute in pregnant women with a clear indication for antiplatelet therapy who are allergic to aspirin. Clopidogrel should only be used when strictly necessary and for the shortest duration possible during pregnancy due to the lack of observational studies in humans [1]. The primary obstetric risk with clopidogrel use is increased risk of intrapartum and postpartum hemorrhage [2]. Its longer duration of action necessitates discontinuation 5–7 days before delivery. However, there is no evidence that clopidogrel increases placental abruption or other antepartum obstetric bleeding events [2]. The other antiplatelet agents are not recommended for use in pregnant women due to the lack of safety data [1,23].

## 4. Role of Anticoagulation Treatment in Pregnancy

**Figure 1:**
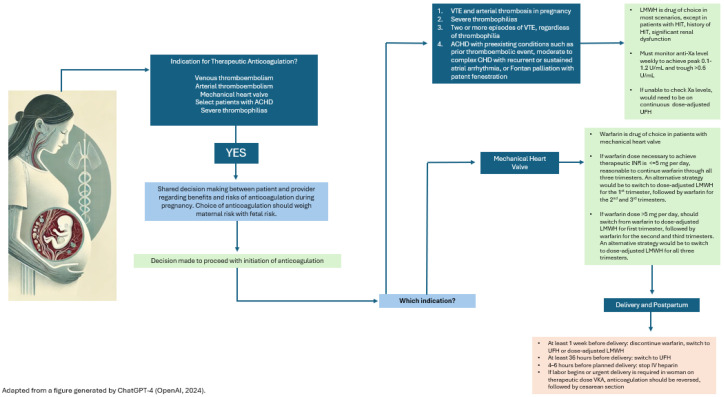
Anticoagulation selection in pregnant women.

Pregnant women are at an increased risk of both venous (~20%) and arterial (~80%) thrombosis due to the hypercoagulable state of pregnancy [4]. There are numerous indications for anticoagulation treatment during pregnancy, including venous thrombosis, arterial thrombosis, and mechanical valves. The preferred anticoagulant in pregnant women is LMWH, as intravenous UFH is logistically challenging to use. Vitamin K antagonists such as warfarin are known teratogens but demonstrate the greatest reduction in thrombotic risk in patients with mechanical heart valves. Selecting the appropriate anticoagulant during pregnancy requires thoughtful discussion between patient and provider that balances therapeutic benefit versus maternal and fetal risks, while accounting for a safe delivery plan [24] (Figure 1).

### 4.1. Venous Thrombosis

Venous thromboembolism (VTE) may manifest during pregnancy as deep vein thrombosis (DVT) or pulmonary embolism (PE). The risk of VTE is increased five-fold in pregnant women compared to non-pregnant women of comparable age and is an important cause of maternal morbidity and mortality. It is also important to recognize that the risk of VTE, predominantly PE, increases during the postpartum period to almost 15- to 35-fold compared to age-matched non-pregnant women [9]. Other risk factors for VTE in pregnancy include hormonally induced increased venous capacitance, decreased venous outflow, mechanical obstruction by the uterus, decreased mobility, and vascular injury. When a DVT occurs during pregnancy, it is more likely to be proximal, massive, and in the left lower extremity [4]. The left-sided predominance is thought to be a May Thurner-like presentation, in which the gravid uterus exacerbates right iliac artery compression of the left iliac vein against the lumbar vertebra as it anatomically crosses over [25].

The treatment of VTE in pregnancy requires careful consideration of both maternal and fetal risks and benefits. There have been no large high-quality studies examining the safety and efficacy of outpatient treatment of VTE during pregnancy, but guidelines are derived from data from the non-pregnant population [4]. LMWH is the drug of choice for treatment of VTE in pregnancy, except in patients with heparin-induced thrombocytopenia (HIT), a history of HIT, or significant renal dysfunction [4]. Adjusted-dose LMWH should continue through pregnancy and, at minimum, for 6 weeks postpartum. A longer duration of therapy may be indicated depending on the timing of VTE during pregnancy, prior VTE history, or the presence of a thrombophilia [7]. Hospitalization for the initiation of anticoagulation therapy may be indicated in cases of hemodynamic instability, large clots, or maternal comorbidities.

Prophylactic LMWH/UFH therapy is recommended for pregnant women with a history of single unprovoked or estrogen-related VTE and/or those with severe thrombophilias, such as antithrombin deficiency, homozygosity for factor V Leiden or Factor II G20210A and double heterozygotes, or women with combined thrombophilias. LMH/UFH at intermediate dose is recommended for those with two or more episodes of VTE, regardless of thrombophilia [7]. The presence of antiphospholipid antibodies (aPLs), triple positivity for lupus anticoagulant (LA), anticardiolipin, and anti B2 glycoprotein antibodies, or LA alone, are recognized as risk factors for both venous and arterial thromboembolism and may also be associated with pregnancy-related morbidity, such as preeclampsia, intrauterine growth retardation, and fetal losses due to placenta malperfusion [26,27]. Prophylactic anticoagulation is not indicated in asymptomatic pregnant women with triple-positive aPLs or any combination that includes the LA during pregnancy but is suggested in the postpartum period [28].

### 4.2. Arterial Thrombosis

Arterial occlusion is classically due to atherosclerotic plaque, with subsequent ischemia and infarction. However, these events are rare in women of childbearing age. Paradoxical emboli through a patent foramen ovale or atrial septal defect; arterial thrombosis due to valvular heart disease, drug-induced vasospasm, or thrombophilia; or atrial thrombosis due to valvular heart disease and/or atrial fibrillation are important alternative etiologies of thromboembolism in young women.

A wide variety of drugs have been reported to cause arterial spasm and/or thrombosis, including ergot derivatives and cocaine [26]. Ergot derivatives, which can be used to control postpartum bleeding, may lead to coronary artery spasm and have been associated with myocardial infarction following delivery or abortion. Pregnant cocaine users may experience myocardial ischemia and infarction because of coronary artery spasm and/or coronary artery thrombosis due to cocaine-induced vasoconstriction of large and small coronary vessels and cocaine-induced endothelial dysfunction [26].

Antiphospholipid syndrome (APS) is associated with significant long-term morbidity and risk of sudden death from major arterial thrombosis. Early recognition and treatment are crucial. The most common site of arterial thrombosis is the cerebral circulation, but coronary, renal, and mesenteric arteries can be involved [26]. Screening should be completed in women with a history of thrombosis and/or fetal loss or severe preeclampsia.

Acute myocardial infarction is rare in women of childbearing age, with an estimated incidence of 5.2 per 1000 in women between 35–44 years of age. In the absence of atherosclerosis, coronary ischemia may be due to coronary spasm or in situ thrombus formation, increased by the hypercoagulability of pregnancy. Definite or probable coronary artery thrombosis without atherosclerosis has been seen in 21% of all pregnancy-associated myocardial infarctions [26]. In terms of management, there are no large high-quality studies demonstrating the safety and efficacy of thrombolytic therapy during pregnancy. Heparin is generally the preferred anticoagulant during pregnancy, along with treatment with low-dose aspirin, nitrates, beta-blockers, and calcium channel blockers [26].

Finally, pregnancy is also associated with an increased risk of stroke. Primary large-vessel occlusion is caused by atherosclerosis, dissection, or arteritis. Primary small-vessel occlusion is seen in patients with arteritis, APS, or eclampsia. Embolic stroke may be caused by arterial thrombosis, paradoxical embolism, or cardiac sources of emboli, such as atrial fibrillation, mural thrombus, or prosthetic valves [26].

Detailed recommendations for the pharmacologic treatment of arterial thrombosis are beyond the scope of this review. From a pregnancy perspective, the mantra of “a healthy mother helps achieve a healthy fetus” is paramount. In situations of myocardial infarction, stroke, or other forms of arterial thrombosis, the choice of anticoagulation versus antithrombotic therapy depends on the specific medical indication, in the context of patient comorbidities and obstetric considerations. However, pregnancy should not prevent the administration of life-saving therapies to the mother.

### 4.3. Prosthetic Valves

Valvular heart disease is a common source of arterial embolism. Worldwide rheumatic valvular disease with resultant mitral stenosis is the most common valvular lesion that affects women of childbearing age [29]. Mitral valve prolapse with secondary mitral regurgitation and bicuspid aortic valve disease with secondary aortic stenosis and/or regurgitation are the next most common valvular lesions [26]. Bicuspid aortic valve, which affects roughly 2% of the population, with up to 50% requiring valve replacement by age 50, disproportionately affects childbearing women in developed countries [30]. In addition, congenital heart disease, with or without repair, is almost always characterized by valvular disease (stenosis or regurgitation) of the left- and right-sided valves.

The incidence of thromboembolism in mitral stenosis is about 1.5–4.5% per year [26]. Risk factors include atrial fibrillation and history of prior thromboembolism. Atrial fibrillation is associated with 7- to 18-fold risk of stroke in these patients [26], due to thrombus formation in the left atrial appendage that travels to the cerebral circulation. Rheumatic mitral stenosis can portend higher rates of atrial fibrillation and thromboembolic disease. Therefore, anticoagulation with warfarin therapy, with or without adjunctive antiplatelet therapy, is often recommended on a case-by-case basis.

For women with prosthetic heart valves, anticoagulation may be indicated depending on the type of valve. About 60% of prosthetic heart valves are mechanical, usually made of titanium or carbon alloy components. The remaining 40% of prosthetic heart valves are bioprosthetic, usually of porcine or bovine origin. Mechanical valves are expected to last at least 30 years, while bioprosthetic valves have a shorter lifespan of about 10–15 years. Patients with bioprosthetic valves are usually maintained on lifelong low-dose aspirin. However, mechanical heart valves are thrombogenic, which necessitates long-term anticoagulation. However, the need to maintain adequate anticoagulation to prevent valve thrombosis must be balanced against the risks of teratogenicity, fetotoxicity, and bleeding [31]. The annual incidence of prosthetic valve thrombosis in the non-pregnant population is 0.1–5.7%, with higher rates observed in those with a mechanical mitral valve prosthesis and/or subtherapeutic anticoagulation [26]. Data show that women with mechanical valves in pregnancy have a higher risk of maternal adverse cardiovascular events, obstetric morbidity such as hemorrhage or preterm birth, and fetal complications, including growth restriction, miscarriage, and stillbirth [32]. Estimated maternal mortality in women with mechanical valves during pregnancy is 1.4% [31].

In general, the presence of a bioprosthetic valve during pregnancy is not an indication for anticoagulation. Anticoagulation with oral VKA is recommended for mechanical valves during pregnancy to prevent thrombotic complications [32,33]. The target INR depends on multiple factors, such as the type of valve and patient comorbidities. Risk factors for higher embolic risk are older types of mechanical valves, specifically Starr–Edwards and Bjork–Shiley valves, particularly in the mitral position, multiple prostheses, history of atrial fibrillation, and left ventricular dysfunction [26]. Factors that are associated with slightly lower thromboembolic risk include newer mechanical valves, particularly the On-X, St. Jude, or Edwards Duromedics, particularly in the aortic position [26]. Patients with a higher thromboembolic risk should target an INR goal of 2.0–3.0; those with lower thromboembolic risk should target an INR goal of 2.5–3.5.

**Figure 2:**
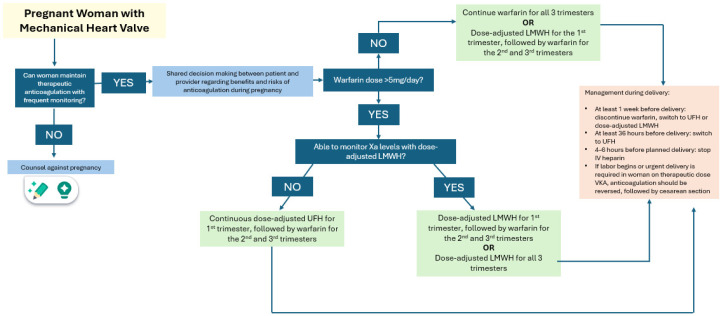
Anticoagulation management of woman with mechanical heart valve. Adapted from [8].

Maternal risk during pregnancy is lowest for women with well-monitored warfarin therapy, but this is associated with greater fetal risk. Exposure to warfarin in the first trimester is associated with warfarin embryopathy, with a dose-dependent effect. Exposure to warfarin in the third trimester increases the risk of fetal hemorrhage during vaginal delivery. To reduce the risk of warfarin embryopathy, women on warfarin therapy who are not intending to conceive should be counseled on the use of reliable contraception, while those who are intending to conceive should perform frequent pregnancy testing and consider switching to dose-adjusted LMWH. The choice of anticoagulation and timing of switching must be made on a case-by-case basis. The current American Heart Association (AHA) and American College of Cardiology (ACC) guidelines [34] recommend that it is reasonable to continue warfarin through all three trimesters of pregnancy if the dose necessary to achieve a therapeutic INR is <=5 mg per day after full risk and benefit discussion between patient and provider (Figure 2). If the dose exceeds 5 mg per day, guidelines suggest that once pregnancy is achieved, warfarin be switched to dose-adjusted LMWH before 6 weeks of gestation through the first trimester [32,33]. Pregnant patients using LMWH are recommended to have anti-Xa levels checked weekly to achieve a peak of 0.7–1.2 U/mL and trough of >0.6 U/mL [7,11]. For the second and third trimesters, it would be reasonable to switch back to warfarin or remain on dose-adjusted LMWH. Guidelines also recommend low-dose aspirin daily in the second and third trimesters for pregnant patients with a mechanical prosthesis [32]. To prevent fetal hemorrhage, warfarin should be avoided close to term. Guidelines recommend discontinuing warfarin at least 1 week prior to delivery and switching to continuous-infusion UFH, or dose-adjusted LMWH with bridging with UFH infusion if needed in the hours prior to delivery. In the postpartum period, therapeutic anticoagulation is restarted when safe with infusion of UFH or LMWH for bridging back to warfarin, which is safe and the preferred choice during breastfeeding [32].

The international Registry of Pregnancy and Cardiac Disease (ROPAC) tracked pregnancy outcomes of 212 patients with a mechanical valve, 134 patients with a tissue valve, and 2620 other patients without a prosthetic valve [31,32]. In these patients, maternal mortality occurred in 1.4% of patients with a mechanical valve, and valve thrombosis occurring in 10 patients with a mechanical valve (4.7%). In five of those patients, thrombosis occurred in the first trimester when patients had been switched to a form of heparin [31]. When transitioning pregnant patients from warfarin to LMWH, care must be taken to ensure that anticoagulation remains therapeutic throughout the transition period. There are emerging data to suggest that both peak and trough values are important for this in pregnant women with mechanical valves [32].

Data show that women with a mechanical valve experience significantly more bleeding complications than women without a mechanical valve. Twenty-three percent of women with a mechanical valve experienced a hemorrhagic event, and most events occurred around the time of delivery. This may be attributed to the higher rates of cesarean deliveries in women with mechanical valves, despite data showing that vaginal delivery is safe in women with cardiac disease. Even in pregnant women with valvular heart disease, guidelines recommend that the mode of delivery should be guided by standard obstetric indications. One exception is women who present in labor on warfarin, where anticoagulation should be reversed, followed by cesarean section to avoid fetal intracranial hemorrhage [32]. Strategies to increase vaginal deliveries when appropriate should help to decrease the rate of maternal hemorrhage. Due to the risks and complexity of anticoagulation management during pregnancy, women with mechanical heart valves should be extensively counseled before and during pregnancy.

## 5. Role of Prophylactic Anticoagulation in Pregnancy

### 5.1. Congenital Heart Disease

Due to advancements in surgical technique and medical treatment in the last decades, increasing numbers of children are surviving into adulthood with congenital heart disease (CHD) [35]. Adults with congenital heart disease (ACHD) are at higher risk of arrhythmias and stroke, but usually lack common risk factors that favor anticoagulation in the acquired heart disease population, such as older age, hypertension, diabetes mellitus, or previous stroke. Establishing clear guidelines for anticoagulation management and thromboembolism prevention in ACHD, particularly during pregnancy, is challenging due to the heterogeneity of the patient population and limited availability of robust clinical evidence, such as randomized controlled trials [15]. Preconception counseling between the patient, an obstetrician, and a cardiologist with expertise in CHD is essential.

Anticoagulation in ACHD may be indicated during pregnancy for preexisting conditions such as atrial arrhythmias, prior thromboembolic events, and mechanical heart valves [15]. Atrial arrhythmias affect almost 50% of the ACHD population and can occur in even simple CHD, such as atrial septal defects [15]. The risk of atrial arrhythmia is highest amongst patients with complex CHD, such as transposition of the greater arteries after atrial switch operation, or those with Fontan palliation [15]. Scoring systems, such as the CHA_2_DS_2_-VASc and HAS-BLED scores, can be used in patients with simple CHD. Prophylactic anticoagulation is generally recommended for prevention of thromboembolism in patients with sustained or recurrent arrhythmia and moderate or complex CHD, such as patients with tetralogy of Fallot or those mentioned above [15]. Additionally, women with CHD may also have an unrepaired or residual intracardiac shunt that can be a source of paradoxical emboli in the hypercoagulable milieu of pregnancy. Indications for anticoagulation may also develop during pregnancy due to the physiologic changes during pregnancy. Cardiac output, heart rate, blood volume, and sympathetic activity all increase in a predictable pattern through pregnancy and increase the risk of atrial arrhythmia. Women with CHD may also have residual valvulopathy, atrial dilatation, previous atrial surgical scar, or ventricular dysfunction, which further enhance their risk of atrial arrhythmia [15].

Fontan palliation is one of the most complex forms of repaired congenital heart disease, with important physiologic sequelae that increase patients’ thrombotic risk and impact maternal and fetal outcomes during pregnancy. Fontan palliation is a multi-step surgical pathway where the systemic venous return is separated from the heart and directed to the pulmonary artery through a surgically created baffle or conduit; oxygenated blood from the pulmonary circulation returns to the common atrium and single ventricle, then is pumped into the systemic circulation. This passive pulmonary blood flow depends on persistent central venous congestion with low left atrial pressure, which increases venous stasis and thrombotic risk. This risk is compounded by the high rates of atrial arrhythmia in patients with Fontan palliation. Slightly older data found that 20% of patients older than 16 years had developed atrial arrhythmia, and 7% of patients had experienced a thromboembolic event (stroke or transient ischemic attack, pulmonary embolism) at a median age of 22.6 years [36]. High central venous pressure also causes chronic liver congestion, with potential synthetic dysfunction or thrombocytopenia, which alters bleeding and thrombotic risk. This system is also preload dependent, with blunted augmentation of cardiac output during exercise [37], which affects how women experience the physiologic changes of pregnancy and delivery planning. Finally, sometimes a fenestration (connection) is created between the venous baffle/conduit and the common atrium as a “pop off” valve, which functions like an atrial septal defect in the normal heart and creates the possibility of paradoxical embolism.

Consequently, patients with Fontan palliation are maintained on at least low-dose aspirin for thromboprophylaxis. Indications for anticoagulation in Fontan patients include history of atrial arrhythmia, as well as previous thromboembolism (cerebrovascular event, transient ischemic attack, pulmonary embolus, venous thromboembolism, or thrombus in Fontan circulation). Compared to older echocardiography data that suggested that thrombus in Fontan circulation may be found in as many as 17–33% of patients after Fontan palliation, more recent MRI data found that only 1/119 patients (0.5%) had an intracardiac thrombus on routine cardiac MRI imaging [38], perhaps due to increased recognition and anticoagulation therapy in Fontan patients. Until the last few years, treatment of thromboembolism in patients with Fontan circulation has predominantly used oral VKA with a usual INR goal of 2.5 (2.0–3.0). However, there is increasing evidence for the use of DOACs in ACHD patients, including Fontan patients [39].

Most patients with Fontan palliation can tolerate pregnancy. However, there is an increased risk of adverse maternal events such as atrial arrhythmias (3–37%), heart failure (3–11%), thromboembolism (5%), antepartum hemorrhage (11%), and postpartum hemorrhage (14%) [40]. Live birth rate in the largest systematic review of pregnancy outcomes in Fontan patients was only 45%, with most pregnancy losses due to miscarriage (45%) [37,38]. There was also an increased risk of fetal complications, such as prematurity (59%), small size for gestational age (20%), and neonatal death (5%) [40]. The likelihood of congenital heart disease is also higher in children of women with CHD (3–5% compared to ~1% in the general population [41].

Therefore, careful preconception counseling should be provided to all women with Fontan palliation, which includes careful medication review, discussion of the maternal and fetal risks during pregnancy, planned monitoring through pregnancy, and a safe delivery plan. Their care during pregnancy should be managed by an experienced multi-disciplinary team at a tertiary care center with maternal–fetal medicine, cardiology with expertise in CHD, anesthesiology, hematology, and neonatology. Delivery (induction of labor) at 37 to 39 weeks of gestation is recommended due to the high risks of maternal and fetal complications [42].

Women who are not on anticoagulation should be at minimum on low-dose aspirin throughout pregnancy for thromboembolism prevention and to decrease preeclampsia risk [42]. It may be reasonable to add prophylactic-dose LMWH throughout pregnancy given the thromboembolic risk and potential for serious sequelae in Fontan patients [42]. There is also some thought that perhaps patients with a patent Fontan fenestration should be considered as higher risk for thromboembolism and considered for therapeutic anticoagulation in addition to low-dose aspirin [42]. Women who are on anticoagulation with either DOACs or oral VKA should be transitioned to dose-adjusted therapeutic LMWH throughout pregnancy with appropriate anti-Xa-level monitoring. Prior to delivery, usually at induction of labor, women will need to be counseled on the timing of LMWH dosing, as placement of neuraxial anesthesia is recommended 12 h after prophylactic anticoagulation or 24 h after therapeutic anticoagulation. Women with Fontan circulation have a lower thrombotic risk, therefore bridging anticoagulation after their last dose of LMWH is usually not required.

Though Fontan physiology is known to be preload dependent, women with Fontan circulation tolerate Valsalva during active labor and can deliver vaginally. An assisted second stage with forceps or vacuum delivery may be helpful for reducing the duration of Valsalva [42]. Additionally, positioning in the left lateral decubitus position can help reduce IVC compression and facilitate venous return. Caesarean delivery is recommended in women who develop intractable heart failure or arrhythmia at the time of delivery, in addition to obstetric reasons [42], with the goal of maintaining preload and blood pressure throughout surgery. Additional considerations during delivery include telemetry monitoring and air filters for preventing paradoxical embolism.

In the postpartum period, patients who have an indication for anticoagulation are usually started on therapeutic-dose intravenous UFH or, more commonly, LWMH 12–24 h after delivery. There should be shared decision-making regarding when to transition back to oral VKA for those who were taking VKA prior to pregnancy, especially for women who also have a mechanical valve. As there are no large observational studies or randomized controlled trials studying the safety of DOACs during breastfeeding, these agents are not recommended at this time, and patients must decide whether they will continue with LMWH or consider oral VKA for the duration of breastfeeding. Women with Fontan palliation who do not require therapeutic anticoagulation should be considered for prophylactic-dose LMWH for at least 6 weeks postpartum, in addition to low-dose aspirin, then transitioned back to daily low-dose aspirin only. This is also an important time for discussion of reliable contraception, such as progestin-only contraception, mechanical options, or sterilization if desired by the patient.

In general, patients with CHD have a higher risk of arrhythmias, thromboembolism, and maternal and fetal morbidity during pregnancy. Fontan patients are particularly at risk, and shared decision-making is important in the management of antithrombotic agents throughout pregnancy that balance maternal and fetal bleeding and thromboembolic risks. Careful preconception counseling is vital, and these patients should be followed in specialized tertiary centers with a multidisciplinary team of cardiologists, obstetricians, hematologists, and anesthesiologists.

### 5.2. Hypercoagulable Disorders

Thrombophilias are a group of inherited or acquired hypercoagulable states that increase thrombosis and are crucial to recognize for pregnancy planning. Inherited thrombophilias include antithrombin III deficiency, protein C and protein S deficiency, activated protein C resistance due to factor V Leiden, and prothrombin gene mutation (factor II G20210A). The most common inherited thrombophilias are factor II G20210A and factor V Leiden, which account for up to 70% of diagnosed inherited thrombophilias [43]. The main acquired thrombophilia is APS. These levels can be modified due to acquired factors, as well as aging [43]. Severe inherited thrombophilias are deficiencies in anticoagulant proteins (antithrombin, protein C and S deficiencies), homozygosity for factor V Leiden or factor II G20210A and double heterozygotes, or women with combined thrombophilias. Severe acquired thrombophilia is triple-positive APS or LA-positive APS. Inherited thrombophilias mainly present with VTE, while acquired thrombophilias are associated with both venous and arterial thrombotic events [43].

In pregnant patients with thrombophilias, anticoagulation initiation should be based on an individualized risk assessment of factors such as personal history of VTE, severity of inherited thrombophilia, family history of VTE, and additional risk factors such as cesarean delivery, obesity, and prolonged immobility [44]. The consensus statement regarding thromboprophylaxis in pregnant women with inherited thrombophilia recommends pharmacologic thromboprophylaxis if the VTE risk is 3% or greater. A 2017 meta-analysis showed that the absolute risk of VTE exceeded 3% in women with antithrombin deficiency, protein C deficiency, protein S deficiency, or homozygosity for factor V Leiden [43]. Ultimately, the decision to initiate prophylactic anticoagulation is complex and requires a thoughtful discussion of the risks and benefits of anticoagulation in each patient.

## 6. Conclusions

The initiation of antiplatelets and anticoagulation in pregnancy can be a challenging decision due to the indications for these agents, along with their adverse effects and dosing complexities (Table 2). This review article highlights the myriad of indications for these agents in the setting of the hypercoagulable state of pregnancy, along with possible adverse effects and dosing recommendations. The decision to initiate these agents during pregnancy should ultimately involve a multidisciplinary team of specialists at a tertiary care center including cardiologists, obstetricians, hematologists, and anesthesiologists. Future studies should include larger populations of pregnant patients to fully analyze the risks and benefits of anticoagulation choices in this population.

## Figures and Tables

**Table 1:** Risk assessment for aspirin initiation for prevention of preeclampsia.

Risk Level	Risk Factors	Recommendation
Low	History of stillbirth History of fetal growth restriction History of early pregnancy loss	No aspirin recommended
Moderate	Nulliparity Obesity (body mass index > 30) Family history of preeclampsia Sociodemographic characteristics (African American race, low socioeconomic status) Age 35 years or older Personal history factors (e.g., low birthweight or small for gestational age, previous adverse pregnancy outcome, more than 10-year interpregnancy interval)	If at least one of these moderate-risk factors is present, low-dose aspirin can be considered
High	History of preeclampsia, especially when accompanied by an adverse outcome Multifetal gestation Chronic hypertension Type 1 or type 2 diabetes Renal disease Autoimmune disease	If at least one of these high-risk factors is present, low-dose aspirin is recommended

Adapted from [5].

**Table 2:** Commonly used antiplatelets and anticoagulants in pregnancy.

Agent	Type of Agent	Mechanism of Action	Primary Indications in Pregnancy	Typical Dosing	Obstetric Risks
Aspirin	Antiplatelet	Inhibits COX-1 and COX-2 in dose-dependent fashion. Anti-inflammatory and anti-platelet properties [1,2,5].	Prevention of preeclampsia Acute coronary syndrome Stroke ACHD: unrepaired shunt lesion, aortic coarctation, moderate to complex CHD Mechanical heart valve in second and third trimesters Bioprosthetic heart valve	Preeclampsia prophylaxis: PO aspirin 81mg/day For preeclampsia, based on US guidelines, initiate between 12 weeks and 28 weeks of gestation (optimally before 16 weeks) and continue daily through delivery	Generally considered safe in pregnancy at low dose Contraindications: aspirin allergy, hypersensitivity to other salicylates and/or NSAIDs, history of nasal polyps, history of aspirin-induced bronchospasm Relative contraindications: gastrointestinal bleeding, active peptic ulcer disease, other sources of gastrointestinal or genitourinary bleeding, severe hepatic dysfunction
Clopidogrel Ticagrelor Prasugrel Cangrelor	Antiplatelet	Inhibits P2Y12, which inhibits platelet aggregation and activation. Clopidogrel and prasugrel are irreversible inhibitors, while ticagrelor and prasugrel are reversible inhibitors. Clopidogrel’s activity is due to its active metabolite [1,2].	Clear indication for antiplatelet therapy but with allergy to aspirin	PO clopidogrel 75 mg/day	Primary risk with clopidogrel is intrapartum and postpartum hemorrhage No safety data available for ticagrelor, prasugrel, and cangrelor use in pregnancy
Warfarin	Anticoagulant (vitamin K antagonist)	Interferes with cyclic interconversion of vitamin K and its 2,3 epoxide [2,6,7,8].	Mechanical heart valve	PO warfarin daily: typical dosing is 2–10 mg, though dose can be outside of this range depending on individual drug metabolism Dosing based on monitoring with INR levels. Usual therapeutic goal range is 2.0–3.0 (variable INR goals depending on indication and individual case)	Crosses the placenta Exposure during first trimester: warfarin embryopathy (dose dependent) Exposure during third trimester: fetal intracranial hemorrhage during vaginal delivery Heightened fetal risk if dose > 5 mg daily. Greatest fetal risk when exposure in first trimester.
Low-molecular-weight heparin (enoxaparin, dalteparin)	Anticoagulant (heparin-based)	Preferentially inhibits factor Xa, and to a lesser extent, factor IIa [2,7,10,45].	Usual treatment of choice for VTE and arterial thrombosis in pregnancy Severe thrombophilias Two or more episodes of VTE, regardless of thrombophilia ACHD with preexisting conditions such as prior thromboembolic event, moderate to complex CHD with recurrent or sustained atrial arrhythmia, or Fontan palliation with patent fenestration	***VTE Prophylaxis***: SQ Enoxaparin and Dalteparin dosed daily. Dose depends upon (1) low vs. intermediate patient risk (2) weight-based adjustment required (3) timing (trimester vs. post-partum) ***VTE Treatment***: SQ Enoxaparin 1 mg/kg BID or SQ Dalteparin 100 units/kg BID Monitored with anti-Xa level Prophylactic, intermediate dose, or adjusted-dose	Generally considered safe in pregnancy: does not cross the placenta Contraindicated in those with HIT, history of HIT, or significant renal dysfunction
Unfractionated heparin	Anticoagulant (heparin-based)	Preferentially inhibits factor IIa (thrombin), potentiating the effect of antithrombin [1,2,7,10,45].	Anticoagulation but in cases of severe renal insufficiency	***VTE Prophylaxis***: SQ Heparin 5000–10,000 units BID depending on trimester, post-partum, weight, and indication. ***VTE Treatment***: therapeutic anticoagulation dosing will be variable with either heparin IV or SQ administration. Monitored with aPTT (note: aPTT will be shortened in pregnancy due to increases in factor VIII) Prophylactic, intermediate dose, or adjusted-dose	Generally considered safe in pregnancy: does not cross the placenta Contraindicated in those with HIT or history of HIT

## Data Availability

No new data were created or analyzed in this study. Data sharing is not applicable to this article.
